# Early literacy and comprehension skills in children learning English as an additional language and monolingual children with language weaknesses

**DOI:** 10.1007/s11145-016-9699-8

**Published:** 2016-10-08

**Authors:** Claudine Bowyer-Crane, Silke Fricke, Blanca Schaefer, Arne Lervåg, Charles Hulme

**Affiliations:** 1 Department of Education, University of York, Heslington, York, YO10 5DD UK; 2 Department of Human Communication Sciences, University of Sheffield, Sheffield, UK; 3 Department of Education, University of Oslo, Oslo, Norway; 4 Division of Psychology and Language Sciences, UCL, London, UK

**Keywords:** EAL, Reading comprehension, Word reading, Oral language

## Abstract

Many children learning English as an additional language (EAL) show reading comprehension difficulties despite adequate decoding. However, the relationship between early language and reading comprehension in this group is not fully understood. The language and literacy skills of 80 children learning English from diverse language backgrounds and 80 monolingual English-speaking peers with language weaknesses were assessed at school entry (mean age = 4 years, 7 months) and after 2 years of schooling in the UK (mean age = 6 years, 3 months). The EAL group showed weaker language skills and stronger word reading than the monolingual group but no difference in reading comprehension. Individual differences in reading comprehension were predicted by variations in decoding and language comprehension in both groups to a similar degree.

## Introduction

Reading development depends upon both decoding and oral language skills, as summarised by the Simple View of Reading (Gough & Tunmer, 1986). Moreover, different cognitive skills have been identified that support the development of different aspects of reading: letter knowledge and phonological processing appear to underpin decoding (e.g. Hulme, Bowyer-Crane, Carroll, Duff, & Snowling, 2012; Lonigan, Burgess, & Anthony, 2000; Lonigan et al., 2009; Muter, Hulme, Snowling, & Stevenson, 2004), while vocabulary and grammar underpin reading comprehension (e.g. Muter et al., 2004; Oakhill, Cain, & Bryant, 2003; Oakhill & Cain, 2012; Ricketts, Nation, & Bishop, 2007). In addition, research suggests that the relative importance of these skills to reading comprehension changes over time, with language skills becoming more predictive of reading comprehension as children gain mastery over decoding skills (e.g. Tilstra, McMaster, Van den Broek, Kendeou, & Rapp, 2009; Vellutino, Tunmer, Jaccard, & Chen, 2007). Much of this research has been carried out with English-speaking children, although there is a growing body of research highlighting similarities and differences in patterns of reading development found in children learning to read in languages other than English (e.g. Babayagit & Stainthorp, 2007; Caravolas et al., 2012; Florit & Cain, 2011; Georgiou, Torppa, Manolitsis, Lyytinen, & Parrila, 2012).

In contrast, less research exists exploring the component processes that underpin the early reading development of children learning to read in English as an additional language (EAL), particularly in a UK context where the language background of English language learners is diverse. Recent statistics from the UK Department for Education indicate that 20.1 % of children in UK primary schools are learning English as an additional language (Department for Education, 2016). Results from national tests of language and literacy reveal a consistent achievement gap in many areas between EAL children and their monolingual English-speaking peers at the early stages of schooling in the UK (Strand, Malmberg, & Hall, 2015). There is clearly a need to provide support for EAL children in developing their early language and literacy skills.

Language support may be particularly important in this group. The level of exposure to English prior to school entry varies in EAL children, but many children will enter school with limited English language skills, particularly in terms of vocabulary knowledge (e.g. Mahon & Crutchley, 2006). As such, while typically developing monolingual English-speaking children can use their existing vocabulary knowledge to support the mapping of newly encountered words in print onto their existing phonological and semantic representations, children learning EAL may be encountering both the spoken and written form of a new word simultaneously. In contrast, while non-phonological oral language skills may be weak in this cohort, phonological skills may be a relative strength for children learning more than one language (e.g. Campbell & Sais, 1995; Kang, 2012; Marinova-Todd, Zhao, & Bernhardt, 2010; McBride-Chang & Kail, 2002) although this finding is not replicated in all studies (e.g. Geva & Zadeh, 2006; Lipka & Siegel, 2007; see Melby-Lervåg & Lervåg, 2014 for a review) and these skills have been shown to develop at a similar rate in monolingual and bilingual children (e.g. Limbird, Maluch, Rjosk, Stanat, & Merkens, 2014). Moreover, it should be noted that the similarity between the languages the children are exposed to may affect the degree to which bilingualism may produce an advantage in phonological processing (e.g. Bialystock, Majumder, & Martin, 2003; Loizou & Stuart, 2003). Nonetheless, this profile of literacy and language skills could lead to strong word reading skills but poorer reading comprehension, a pattern that has been found in the literature (Babayigit, 2014, 2015; Burgoyne, Kelly, Whiteley, & Spooner, 2009; Burgoyne, Whiteley, & Hutchinson, 2011, 2013; Hutchinson, Whiteley, Smith, & Connors, 2003; Lesaux, Crosson, Kieffer, & Pierce, 2010) although not consistently (e.g. Lesaux, Rupp, & Siegel, 2007; Lesaux & Siegel, 2003; Limbird et al., 2014). Moreover, Manis, Lindsey, and Bailey (2004) suggested that differences in reading comprehension may not be apparent at the early stages of learning to read in a second language (L2). Their sample of young Spanish-speaking bilinguals showed similar levels of performance on measures of Spanish and English reading comprehension at US Grade 2 and only slight differences in performance at Grade 1.

Research exploring reading development in monolingual and bilingual children has found similar predictors of decoding in both groups (e.g. Chiappe & Siegel, 2006; McBride-Chang & Kail, 2002; Muter & Diethelm, 2001). Similarly, as with monolingual children, research looking at predictors of reading comprehension in bilingual children has found oral language to be a significant predictor of reading comprehension in most studies (e.g. Babayigit, 2014, 2015; Lesaux, Crosson, Kieffer, & Pierce, 2010; Lesaux et al., 2007; Proctor, Carlo, August, & Snow, 2005). Indeed, Kieffer (2012) carried out an analysis of longitudinal data collected from Spanish-speaking English language learners in the US and found that English productive vocabulary was the strongest predictor of English reading comprehension when compared to other measures of oral language. However, not all of these studies directly compared second language learners with their monolingual peers (e.g. Kieffer, 2012; Lesaux et al., 2010; Proctor et al., 2005) and as such have not explored the relative importance of oral language to reading comprehension in children from different language backgrounds. Some studies have found that oral language is a stronger predictor of reading comprehension in second language learners compared to monolingual children although the majority of these have not been carried out in the UK (e.g. Droop & Verhoeven, 2003; Lervåg & Aukrust, 2010; Limbird et al., 2014). In one recent UK study, Babayigit (2014) found that oral language was a significant predictor of reading comprehension in both monolingual and bilingual English-speaking children. However, she also found a marginally stronger association between oral language and reading comprehension in the bilingual children in her sample. Conversely, in a later study, Babayigit (2015) found no significant difference in the strength of association between oral language and reading comprehension, although there was a tendency for this association to be stronger in the EAL group. More studies are needed that directly compare the development of EAL and monolingual children in a UK context. Similarly the majority of these studies include children aged 5 years and over at the first point of testing, with many studies in the UK focusing on children in Year 2 and above (Babayigit, 2014, 2015; Burgoyne et al., 2009, 2011, 2013; Hutchinson et al., 2003). Children begin reading instruction in the UK at approximately 4 years of age. It is important to note that in the UK there is considerable emphasis on systematic phonics instruction at the early stages of learning to read, precipitated by the publication of the Independent Review of the Teaching of Early Reading (Rose, 2006) and reinforced by the introduction in 2012 of a statutory check of decoding skills for all 6-year olds in UK primary classrooms (Department for Education, 2012a). Research suggests that instructional practices directly influence the cognitive skills children utilise during reading (McGeown, Johnston & Medford, 2012). More research is therefore needed investigating early reading and comprehension skills in this cohort within the context of a phonics led curriculum of early reading instruction in UK classrooms.

Recent reports suggest that a large proportion of monolingual English-speaking children are also starting school in the UK with poor oral language (e.g. Bercow, 2008; Law, Todd, Clark, Mroz, & Carr, 2013; Lee, 2013). These children are at risk of difficulties with literacy development (see Pennington & Bishop, 2009 for a review) and educational underachievement much like their EAL peers. However, the nature and aetiology of their language difficulties may be different. While some children learning EAL will have existing language impairments, for many their weaknesses in English may be largely attributed to the challenge of learning a second language. Conversely, the problems facing monolingual children with language weaknesses may be attributed to other factors such as a language delay, a language impairment and/or low socio-economic status (SES; e.g. Clegg, Law, Rush, Peters, & Roulstone, 2015). These children form an important comparison group since EAL children are often put on the Special Educational Needs register (Stow & Dodd, 2003; Sullivan, 2011) and may receive similar instruction and intervention to their monolingual peers with language weaknesses whether or not they have underlying language problems. Given the difference in aetiology of the language difficulties of many EAL children, this may not be appropriate, and it is important to tease these groups apart in order to ensure children receive the most appropriate support.

Research has shown that instructional approaches that combine letter knowledge and phoneme awareness are effective for promoting the development of word level reading (Bowyer-Crane et al., 2008; Lundberg, 1994; Torgesen et al., 1999) whereas teaching to promote broader oral language skills is effective in improving reading comprehension skills (Bianco et al., 2010; Borstrom & Elbro, 1997; Clarke, Snowling, Truelove, & Hulme, 2010; Fricke, Bowyer-Crane, Haley, Hulme, & Snowling, 2013). However, this research typically targets native speakers and only a small number of studies focus on ameliorating the language difficulties of bilingual children, particularly in the UK (see Murphy, 2015 for a review). Understanding the similarities and differences in literacy development between EAL children and their monolingual peers with language weaknesses will help to inform the development of effective literacy instruction and intervention approaches for all children.

In this paper we report data collected in a randomised controlled trial that aimed to evaluate the effectiveness of an oral language intervention programme for EAL children (Schaefer, Fricke, Bowyer-Crane, Millard & Hulme, 2015). Here, we present longitudinal analyses examining the predictors of reading comprehension skills at the end of Year 1 in UK primary school, from measures taken at school entry (UK Reception). Half of the children were learning to read in EAL and half were monolingual peers with language weaknesses (ML). Our first aim was to look at the similarities and differences in the early reading and language skills of these two groups. Our second aim was to examine the similarities and differences in predictors of reading comprehension and word reading skills across the two groups.

## Method

### Participants

We recruited participants from 10 primary schools in the South Yorkshire area. Of the 10 schools selected, 8 had a higher percentage of children eligible for free school meals than the UK national average in the year they were recruited (Department for Education, 2012b). We screened all of the children in the Reception class of these schools at school entry and selected 160 children to participate in a randomised controlled trial evaluating the effectiveness of an oral language intervention. We selected children showing the weakest language skills in relation to their classroom peers based on their performance at the beginning of the project on two subtests from the Clinical Evaluation of Language Fundamentals Preschool II UK (CELF-Preschool II UK; Semel, Wiig, & Secord, 2006a); Expressive Vocabulary and Sentence Structure, and the Non Word Repetition subtest from the Early Repetition Battery (ERB; Seeff-Gabriel, Chiat, & Roy, 2008). 80 children (44 male) were monolingual English-speaking (ML) and 80 children (50 male) were learning EAL. We randomly allocated half of the children to receive oral language intervention (*n* = 40 ML/40 EAL) and half to a waiting control group (*n* = 40 ML/40 EAL). We excluded children with no functional English skills from the sample. In the EAL group, 13.75 % of children were reported to have Urdu as their first language (*n* = 22), and 10 % were reported to be Punjabi speakers (*n* = 16). The remaining children spoke Arabic (*n* = 3), Bengali (*n* = 3), Chinese (*n* = 3), Czech (*n* = 6), French (*n* = 1), Karen (*n* = 2), Kurdish (*n* = 3), Lingala (*n* = 1), Malayalam (*n* = 2), Mandarin (*n* = 2), Pashto (*n* = 6), Polish (*n* = 3), Portuguese (*n* = 2), Romany (*n* = 2), Tamil (*n* = 1), and Vietnamese (*n* = 1), while the official home language of one child was unknown. At the initial test phase, the two groups did not differ in age, *t*(158) = 0.63, *p* = .53, or nonverbal IQ, *t*(158) = −1.03, *p* = .305; see Table 1.

**Table 1:** Means (standard deviations) of EAL and ML groups on measures of literacy, phonological skills, language and nonverbal IQ at t1, and literacy, phonological skills, and language at t2

Measure	T1 reliability	T2 reliability	ML		EAL		Cohen’s *d* ^a^	
			Time 1 n = 80	Time 2 n = 73	Time 1 n = 80	Time 2 n = 71	Time 1	Time 2
Age mths (*t*1)	–		55.51 (3.34)	–	55.18 (3.47)	–	0.01	–
*Oral language*								
CELF Expressive Vocabulary	.82^b^	.84^b^	12.89 (5.76)^e^	20.55 (7.16)^f^	8.31 (6.73)^e^	16.68 (8.52)^f^	0.73	0.52
CELF Sentence Structure	.78^b^	.70^b^	8.60 (3.58)^e^	18.0 (3.52)^f^	7.41 (3.98)^e^	16.73 (3.84)^f^	0.31	0.34
APT Grammar	.88^b^	–	17.91 (5.66)	25.84 (4.18)	13.18 (7.29)	23.80 (4.18)	0.72	0.49
APT Information	.90^b^	–	24.16 (5.52)	31.45 (3.14)	18.58 (7.65)	30.03 (4.10)	0.83	0.39
Listening Comprehension	.78^b^	.69^b^	2.13 (1.73)	5.88 (2.50)	1.68 (1.70)	5.83 (2.55)	0.26	0.02
*Phonological skills*								
ERB Non-word Repetition	.89^b^	–	10.42 (3.59)	–	9.08 (4.10)	–	0.35	
YARC Sound Isolation	.88^b^	–	3.15 (2.94)	–	3.81 (3.48)	–	0.21	–
*Literacy*								
YARC Letter Sound Knowledge	.95^b^	–	8.35 (3.26)	–	9.41 (3.61)	–	0.31	–
YARC Early Word Reading	.98^b^	–	2.41 (3.83)	–	3.93 (5.74)	–	0.31	–
DTWRP Total	–	.99^b^	–	28.71 (18.93)	–	38.58 (19.24)	–	0.52
YARC Reading Comprehension^g^	–	.83^b^	–	7.91 (3.07)	–	8.66 (3.14)	–	0.24
YARC Reading Errors^g^	–	.80^c^	–	15.58 (15.54)	–	9.63 (10.36)	–	0.46
Invented Spelling	.94^b^	–	9.78 (10.39)	64.37 (19.78)	12.11 (12.57)	72.24 (16.18)	0.20	0.43
Phonics Screening Check^h^	–	–	–	26.44 (12.55)	–	32.09 (9.05)	–	0.55
WPPSI Block Design	.84^d^	–	20.13 (2.87)	–	20.79 (4.09)	–	0.16	–

Where different tests are given at t2, reliabilities are reported for both t1 and t2 measures

*WPPSI* Wechsler Preschool and Primary Scale of Intelligence, *YARC* York Assessment of Reading for Comprehension, *DTWRP* Diagnostic Test of Word Reading Processes

^a^Cohen’s d (pooled sd) illustrates the size of the difference between groups at each time point; ^b^ Cronbach’s alpha; ^c^ correlation between parallel test forms; ^d^ split half reliability; ^e^ Clinical Evaluation of Language Fundamentals Preschool II^UK^; ^f^ Clinical Evaluation of Language Fundamentals 4; ^g^ ML n = 65, EAL n = 64; ^h^ ML n = 63, EAL n = 66

### Tests and procedures

Full details of the screening and testing procedures for the intervention study can be found in Schaefer et al. (2015). We gave children a large battery of tests at each time point but only include the measures appropriate to the current research questions in this paper. We collected the data analysed in this paper at two time points; Time 1 (t1) data in the Autumn term of the children’s first year of formal schooling (UK Reception), and Time 2 (t2) data in the Summer term of Year 1, at the end of the children’s second year of formal schooling. We saw children individually on each occasion and trained research assistants administered the measures. We used raw scores in all analyses.

#### Language skills

Listening comprehension (t1, t2)—measured at t1 using a short story from the York Assessment of Reading for Comprehension (YARC; Snowling et al., 2009) which children listened to via headphones and then answered a set of comprehension questions. The maximum score on this task was 8. We used the Understanding Spoken Paragraphs subtest from the Clinical Evaluation of Language Fundamentals 4th Edition (CELF-4 UK; Semel, Wiig, & Secord, 2006b) at t2. This measure has a maximum score of 15.

Expressive Grammar (t1, t2)—measured using the Action Picture Test (APT; Renfrew, 2003). Children are asked questions about a series of pictures that elicit different grammatical constructs in response (e.g. “What is the girl doing?”; “What is the mother going to do?”; “What has the cat just done?”). Children receive a score for grammatical complexity with a maximum possible score of 40. We also gave children the Sentence Structure subtest from the Clinical Evaluation of Language Fundamentals in which they are asked to point to the picture that matches a spoken sentence. We used the subtest from the CELF Preschool II UK (Semel et al., 2006a) scale at t1 which has a maximum score of 22. At t2 we used the CELF-4 UK (Semel et al., 2006b) which has a maximum score of 26.

Expressive Vocabulary (t1, t2)—measured using the CELF Expressive Vocabulary subtest in which children are asked to name a series of pictures. At t1 we used the subtest from the CELF Preschool II UK (Semel et al., 2006a) scale which has a maximum score of 40. At t2 we used the CELF-4 UK (Semel et al., 2006b) which has a maximum score of 54. We included the APT Information score as a measure of vocabulary with children’s responses scored according to the vocabulary used to describe the pictures (max. raw score 40).

#### Literacy skills

Letter Sound Knowledge (t1)—measured using the York Assessment of Reading for Comprehension (YARC)—Early Reading (Hulme et al., 2009) Letter Sound Knowledge subscale in which children are asked to provide the sounds of a series of single letters and digraphs. At t1 we used the core subscale which has a maximum score of 17.

Invented Spelling (t1, t2)—measured by giving children pictures to name and spell. At t1 we showed children five pictures, while at t2 we extended this to 10 pictures. We scored responses for number of consonants correct.

Word Reading (t1, t2)—At t1 we used the Early Word Recognition subtest from the YARC—Early Reading (Hulme et al., 2009) to assess word reading. This measure included 15 regular and 15 irregular words, and had a maximum score of 30. At t2 we measured word reading using the Diagnostic Test of Word Reading Processes (DTWRP; Forum for Research into Language and Literacy, 2012). Three subscales are given on this test—a measure of non-word reading, a measure of exception word reading and a measure of regular word reading. A maximum score of 30 is available for each subscale. We created a composite score of all three measures with a maximum score of 90.

Reading Comprehension (t2)—measured using the York Assessment of Reading for Comprehension (YARC)—Passage Reading (Snowling et al., 2009). We administered the Beginner passage and Level 1 from Form A of the YARC. Beginner passages employ a shared reading paradigm where the experimenter and the child take turns in reading sentences while Level 1 passages are read solely by the child. Following the reading of each passage, children are asked a set of comprehension questions. We created a composite score by combining the scores across the two passages with a maximum score of 16. This measure also provides a Text Reading Accuracy score in terms of the number of errors each child makes during reading.

#### Phonological skills

Phonological Processing (t1)—measured using the Non-Word Repetition task from the ERB (Seeff-Gabriel, Chiat, & Roy, 2008). The maximum score on this task is 18.

Sound Isolation (t1)—measured using the YARC—Early Reading (Hulme et al., 2009) Sound Isolation subscale in which children are asked to repeat a word and then isolate a sound from the beginning, or end of the word. Children are awarded one point for each correct response with a maximum score of 12.

#### General cognitive abilities

Non-Verbal IQ (t1)—measured using the Block Design task from the Wechsler Preschool and Primary Scale of Intelligence (WPPSI; Wechsler, 2003). In this test children are given a set of blocks which they have to arrange to match a series of geometric patterns.

#### Statutory measure of achievement

Phonics Screening Check (Department for Education, 2012a)—this is a statutory measure of phonic decoding administered annually when children are in Year 1. Children are asked to read a list of real words and non-words (maximum score = 40).

## Results

We provide means and standard deviations for measures of language, literacy, and phonological skills at t1 and t2 in Table 1 along with Cohen’s *d* calculated using the pooled standard deviation to show the effect size of the difference between groups at each time point. We used raw scores in all analyses since norms are not available for children learning EAL on the majority of these measures. However, the YARC Reading Comprehension normative sample did include approximately 14 % of children learning EAL, and we provide standard scores for this measure in Table 2. We first looked at the similarities and differences between groups at t1 when children entered UK Reception and before they had received formal reading instruction.

**Table 2:** Mean (SD) standard YARC reading comprehension scores for ML and EAL groups

	ML (n = 65)	EAL (n = 64)
YARC Beginner Passage (Form A)	84.82 (5.03)	85.88 (6.11)
YARC Level 1 Passage (Form A)	81.03 (3.79)	83.98 (5.66)

*YARC* York Assessment of Reading for Comprehension, *ML* monolingual, *EAL* English as an additional language

Independent samples *t* tests showed no significant differences between EAL and ML children on a measure of phonological awareness (i.e. YARC Sound Isolation), *t*(153.65) = −1.30, *p* = .195, or on the Invented Spelling task, *t*(158) = −1.28, *p* = .202. However, ML children outperformed EAL children on measures of language, that is CELF-Preschool II UK Expressive Vocabulary, *t*(154.34) = 4.62, *p* = .000, CELF-Preschool II UK Sentence Structure, *t*(158) = 1.98, *p* = .049, APT Information score, *t*(143.77) = 5.30, *p* = .000, and APT Grammar score, *t*(148.84) = 4.58, *p* = .000) with the exception of performance on Listening Comprehension, *t*(158) = 1.66, *p* = .100. In contrast, EAL children outperformed monolingual children on measures of phonological processing (i.e. ERB Non-Word Repetition), *t*(156) = 2.19, *p* = .030, and word reading (i.e. YARC Early Word Reading), *t*(137.86) = −1.98, *p* = .050. When the latter measure was divided into regular and exception words, a significant difference between groups was only found for exception word reading, *t*(101.25) = −2.24, *p* = .027 with no difference between groups found for regular words, *t*(149.97) = −1.64, *p* = .10.

At the end of Year 1 after 2 years of formal literacy instruction EAL children showed better word reading skills than their monolingual peers (ML) as measured by a composite score of all three subscales of the DTWRP*, t*(142) = 3.10, *p* = .002, made fewer errors when reading text (i.e. YARC Text Reading Accuracy), *t*(111.70) = 2.56, *p* = .012 and had stronger spelling skills as measured by the Invented Spelling task, *t*(142) = −2.61, *p* = .010. They also showed significantly stronger outcomes on the Phonics Screening Check, *t*(112.44) = −2.94, *p* = .004, administered at the end of Year 1. However, the ML children continued to show stronger oral language skills than the EAL group on the majority of measures, that is, CELF 4 UK Expressive Vocabulary, *t*(142) = 2.96, *p* = .004, CELF-4 UK Sentence Structure, *t*(142) = 2.07, *p* = .041, APT Information score, *t*(131.33) = 2.34, *p* = .020, and APT Grammar score, t(142) = 2.92, *p* = .004). The groups did not differ on measures of listening comprehension (i.e. CELF-4 UK Understanding Spoken Paragraphs)*, t*(142) = 0.108, *p* = .914, or reading comprehension (i.e. YARC-Passage Reading)*, t*(127) = 1.37, *p* = .174.

**Fig. 1:**
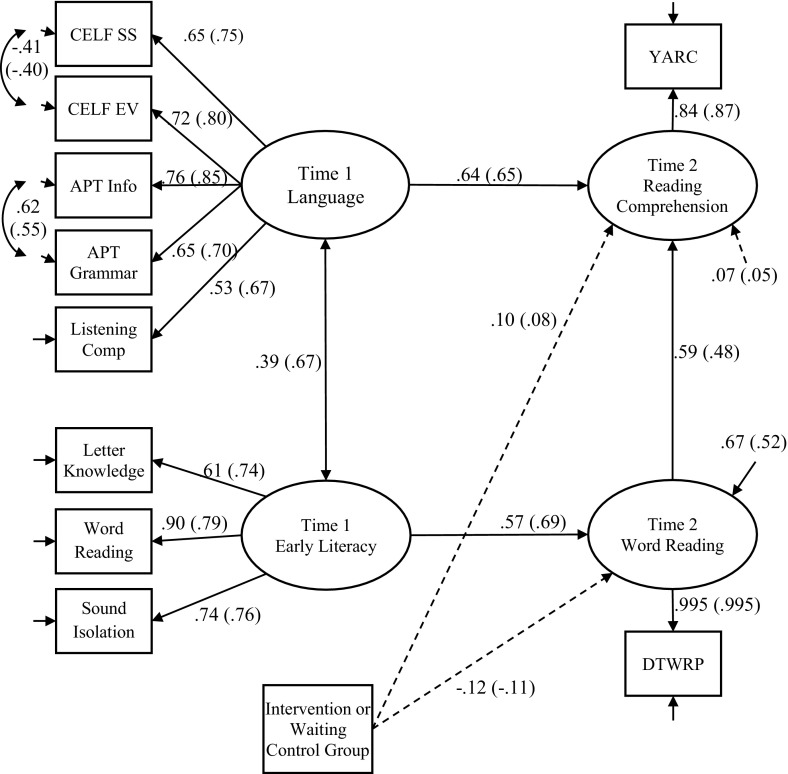
Model showing the prediction of language and early literacy at time 1 on reading comprehension and word reading at time 2 in monolingual children and EAL children (in *parentheses*). The model shows the standardized solution with the exception of the paths from Intervention or Waiting Control Group which show the y-standardized solution as *dashed lines*

Finally, we looked at longitudinal predictors of reading comprehension and word reading in the ML and EAL groups. Initial Pearson correlations for all t1 measures with t2 Reading Comprehension and t2 Word Reading are shown in Table 3. In the ML group, t2 Word Reading processes correlate with t1 early literacy and phonological awareness measures but not with t1 language, while in the EAL group t2 Word Reading correlates with all t1 language and literacy measures. T2 Reading Comprehension correlated with all t1 language and literacy measures in both groups with the exception of Letter Sound Knowledge and Early Word Reading in the ML group. Our principal interest was to trace possible causal influences from early variations in language and emergent literacy skills to variations in later word reading and reading comprehension skills. For this purpose, a two-group structural equation model was constructed (see Fig. 1) using Mplus 7.4 (Muthén & Muthén, 1998–2015) with missing data being handled with full information maximum likelihood estimation. Before creating the two-group structural equation model we established that strong (scalar) measurement invariance was present for the two latent variables in the model since constraining the unstandardized factor loadings and intercepts to be equal across groups resulted in no significant change in fit against the unconstrained model, Δ*χ* ^2^(12) = 17.484, *p* = .132. There was also invariance between the residual covariances that we estimated to take account of common test-specific variance in the CELF and APT tests, Δ*χ* ^2^(2) = 4.584, *p* = .101. Further, since Reading Comprehension and Word Reading were measured as single observed variables, we adjusted for the measurement error (Cole & Preacher, 2014).

**Table 3:** Pearson correlations between performance on t1 early literacy and oral language measures with t2 Word Reading and t2 Reading Comprehension

Time one measures	t2 Reading Comprehension (YARC)		t2 Word Reading (DTWRP)	
	ML	EAL	ML	EAL
CELF Expressive Vocabulary	0.44***	0.38**	0.21	0.34**
CELF Sentence Structure	0.50***	0.50***	0.16	0.36**
APT Grammar	0.46***	0.39**	0.20	0.32**
APT Information	0.43***	0.47***	0.14	0.35**
Listening Comprehension	0.45***	0.42**	0.22	0.36**
ERB Non-Word Repetition	0.41**	0.39**	0.53***	0.46***
YARC Sound Isolation	0.42***	0.39**	0.46***	0.54***
YARC Letter Sound Knowledge	0.17	0.44***	0.47***	0.48***
YARC Early Word Reading	0.14	0.41**	0.38**	0.44***
Invented Spelling	0.43***	0.60***	0.53***	0.52***

*CELF* Clinical Evaluation of Language Fundamentals Preschool II^UK^, *APT* Action Picture Test, *ERB* Early Repetition Battery, *YARC* York Assessment of Reading for Comprehension, *DTWRP* Diagnostic Test of Word Reading Processes

* *p* < .05; ** *p* < .01; *** *p* < .001

We wished to assess the possibly separable influences of language and emergent literacy skills on later word reading and reading comprehension skills. In the model in Fig. 1, we used five language measures to define a Language factor (CELF Preschool Sentence Structure, CELF Preschool Expressive Vocabulary, APT information, APT grammar and Listening Comprehension), and three measures of early literacy skills (YARC Letter Sound Knowledge, YARC Early Word Reading and YARC Sound Isolation) to define a separate Early Literacy factor at t1. We regressed Word Reading scores (DTWRP) and YARC Reading Comprehension scores at t2 on both these two factors measured at t1. We also regressed the t2 measures (DTWRP scores and YARC Reading Comprehension scores) on a dummy coded variable representing the difference between the intervention and control groups. In addition, we regressed YARC Reading Comprehension on Word Reading scores (DTWRP) measured at t2 since theoretically, reading comprehension is expected to be dependent upon levels of word reading skill. As this model was not significantly different from a comparable model where Word Reading scores (DTWRP) at t2 were not regressed on Language at t1 and YARC Reading Comprehension at t2 was not regressed on Early Literacy at t1, Δ*χ* ^2^(4) = 1.259, *p* = .868, we dropped these regressions in the final model. In this final model, all unstandardized covariances and regression coefficients were constrained to be equal across groups. There was no difference between this model and a comparable model with the regression coefficients estimated freely across groups, Δ*χ* ^2^(5) = 2.963, *p* = .706, implying that the predictive pattern does not differ across groups. The covariance between Language and Early Literacy was, however, significantly stronger, Δ*χ* ^2^(1) = 7.686, *p* = .006, among the EAL children compared to the monolinguals.

Figure 1 shows standardized path weights for both the EAL and ML groups (coefficients for the ML group outside the parentheses and for the EAL group inside the parentheses; these standardized coefficients differ slightly between groups due to differences in variance between the groups). In addition, the y-standardized regression coefficients for the dummy coded intervention group variable on t2 Reading Comprehension and t2 Word Reading are shown in order to identify whether changes in these skills occurred as a result of treatment. These y-standardized coefficients are equivalent to Cohen’s *d* (the difference in mean scores between groups in *z*-score units). These coefficients are small in magnitude and nonsignificant, confirming that the intervention did not produce any reliable changes in Reading Comprehension or Word Reading skill at t2.

At t1, the structural model consists of two correlated latent variables (Early Literacy and Language). Early Literacy at t1 predicts Word Reading at t2. In addition, as expected, Language at t1 and Word Reading ability at t2 are both strong predictors of Reading Comprehension at t2. The model accounts for 95 % of the variance in Reading Comprehension skills at t2 for the EAL group and 93 % of the variance in the ML group (the variance explained in reading comprehension in a comparable model where measurement errors were not corrected was 72 and 67 % for the EAL and ML group respectively). The standardized indirect path from Early Literacy at t1 to Reading Comprehension at t2 through Word Reading at t2 is 0.33 and 0.34 for the EAL and ML groups respectively (*p* < .001 for both groups).

The model fits the data well, *χ* ^2^(97) = 116.447, *p* = .087, Root Mean Square Error of Approximation (RMSEA) = 0.050 (90 % CI = 0.000, 0.081), Comparative Fit Index (CFI) = 0.97, Tucker–Lewis Index (TIL) = 0.97, confirming that the structure of the underlying abilities specified in the measurement model is appropriate.

## Discussion

In this paper we investigate the early language and literacy skills of a group of 160 children; 80 monolingual English-speaking children with language weaknesses and 80 children learning to read in EAL. We report data collected at school entry and again after roughly 2 years of formal literacy instruction in the UK. The aim of the paper was first to compare the early reading and language skills of these two groups of children at school entry and after 2 years of formal reading instruction. A second aim was to examine the predictors of Word Reading and Reading Comprehension from school entry to the end of Year 1 across groups.

Comparing their literacy, phonological, and language skills, at both t1 and t2, the monolingual children had significantly better expressive language skills than their EAL counterparts. This is unsurprising at t1 given that the level of exposure to English will vary considerably in the EAL cohort prior to school entry. However, the consistent gap evident at t2 demonstrates that EAL children continue to lag behind in their English oral language skills even after approximately 2 years of formal schooling and when compared to a group of monolingual peers selected as having poor language skills. Conversely, the EAL cohort outperformed the monolingual children on measures of word reading and spelling at both time points, a finding that is not unusual in the literature. For example, Burgoyne et al., (2009) found stronger word reading and text reading accuracy in their EAL participants compared to the monolingual peers. In terms of phonological skills, a phonological processing advantage was found for EAL children on a measure of non-word repetition in this study, which may partly explain the superior word level reading skills of the EAL group. In contrast, there was no significant difference in phonological awareness skills between the EAL and ML groups, which is inconsistent with previous research (e.g. Campbell & Sais, 1995; Kang, 2012; Marinova-Todd et al., 2010; McBride-Chang & Kail, 2002).

However, a number of factors are important to bear in mind when interpreting these findings. First, not all studies have found an advantage for second language learners in phonological skills. Lipka and Siegel (2007) found an advantage for L1 learners on measures of non-word repetition and phonological awareness at kindergarten, and no difference between groups at Grade 3, while Geva and Zadeh (2006) found no difference between L1 and L2 learners on measures of phonological awareness although L2 learners were faster at a rapid automatized naming task. In fact, Melby-Lervåg and Lervåg (2014) only found small and nonsignificant differences between first and second language learners in phonological awareness, which were unaffected by correcting for publication bias. Moreover, the EAL sample in this study was heterogeneous in terms of the languages spoken and therefore the degree of similarity between children’s L1 and English varied greatly across the sample. Bialystock et al. (2003) found that Spanish-English bilinguals performed better than monolingual English speakers and Chinese-English bilinguals on a measure of phoneme segmentation. One interpretation of these findings was that the similarity in phonological structure between Spanish and English facilitated phonological awareness skills in that group; a facilitatory effect not available to the Chinese-English bilinguals due to differences in the structures of the two languages. Similarly, Loizou and Stuart (2003) found that while English-Greek bilingual children outperformed their English monolingual peers on measures of phonological awareness, no such advantage was found for Greek-English bilinguals compared to their Greek monolingual peers. They explained this finding in terms of the relative complexity of the L2 compared to the L1 (i.e. English-Greek children are learning a second language (Greek) with a far simpler phonological structure than their L1 (English), which facilitates their phonological awareness in their L2). In contrast, Greek-English children are learning a second language (English) with a far more complex phonological structure than their L1 (Greek) and as such, gain no advantage in their phonological development from exposure to two languages. Given the heterogeneity in our sample, the relative phonological complexity of the first language of our participants will vary, and this may have a masking effect on any phonological awareness advantage to be found at the group level. Finally, all of the children in the study received systematic phonics instruction in class, including work on phonological awareness, which may have attenuated the expected differences in phonological awareness between the two groups.

An unexpected finding was the lack of any difference in reading comprehension between groups. Many studies have found that children learning EAL demonstrate lower levels of reading comprehension than their monolingual peers (Babayigit, 2014, 2015; Burgoyne et al., 2009, 2011; Hutchinson et al., 2003; Lesaux et al., 2010), a finding supported by a recent meta-analytic review (Melby-Lervåg & Lervåg, 2014). However, it is important to note that we selected the monolingual group in this study as having weak oral language in relation to their peers and they were performing in the low average range on the reading comprehension measure as seen in Table 2. Standard scores for the EAL sample also indicate that they show poor performance on this measure of reading comprehension despite having adequate word reading skills.

It is also of note that we measured reading comprehension in this study using a single comprehension measure. Studies have shown that reading comprehension assessments can tap different skills (e.g. Bowyer-Crane & Snowling, 2005; Keenan, Betjemann, & Olson, 2008; Kendeou, Papadopolous, & Spandouis, 2012; Spooner, Baddeley, & Gathercole, 2004; Melby-Lervåg & Lervåg, 2014) and as such, a more reliable index of reading comprehension should be obtained by using more than one measure (e.g. Droop & Verhoeven, 2003). However, due to the extensive battery of measures given as part of the main RCT, we were only able to include one measure of reading comprehension in this study. Moreover, the children in this study are still at a very early stage in terms of reading comprehension development, a fact reflected in the use of a shared reading paradigm for half of the task to make it more accessible. As mentioned earlier, research shows that differences in the relative value of decoding and linguistic comprehension to the prediction of reading comprehension appear over time (e.g. Tilstra et al., 2009; Vellutino et al., 2007). As such, different findings may occur when looking at reading comprehension at a later point.

The results of our latent variable models did not support the findings from previous studies that oral language is a stronger predictor of reading comprehension in bilingual children (e.g. Babayigit, 2014; Lervåg & Aukrust, 2010; Limbird et al., 2014). In our model, Language at t1 predicted Reading Comprehension at t2 in both ML and EAL learners, and there was no difference in the strength of association between the latent and observed variables across groups. Similarly, Early Literacy at t1 was a significant predictor of Word Reading at t2 in both groups. These findings may be partly attributable to the developmental stage of the children and the fact that the monolingual children in this study had language weaknesses. According to the Simple View of Reading (Gough & Tunmer, 1986), at the early stages of learning to read, reading comprehension will largely be driven by decoding skills with language comprehension becoming more important over time. The children in this study were still at a relatively early stage in their reading development and therefore, their comprehension skills will be largely dependent on their decoding skills. Moreover, given their language weaknesses, these children may be restricted in using their language skills to support their reading comprehension skills. The children in the Babayigit (2014) study in contrast were approximately 9 years of age and therefore at a stage in development where oral language would be expected to play a more significant role in reading comprehension and as such, the predictive strength of these skills may vary. More longitudinal research is needed tracking the reading and language development of children learning EAL and their monolingual peers from school entry to later primary school in order to further investigate these models.

These findings have implications for supporting the reading development of children learning EAL and monolingual children with language weaknesses. Children learning EAL seem to acquire word level reading skills more readily than their monolingual peers with language weaknesses as evidenced by performance on both a standardized measure of word reading and the national phonics screening check. Both groups of children appeared to struggle with reading comprehension. However, the mediation analyses indicated that there was no difference in the strength of association between early oral language and emergent literacy skills, and either word reading or reading comprehension between these two groups. As such, when considering interventions to support reading comprehension for both children learning EAL and monolingual children with language weaknesses, it is important to focus on supporting their oral language skills. However, particular attention needs to be paid to the reading comprehension skills of EAL children from the early stages of learning to read as their word reading skills may mask the identification of their reading comprehension difficulties until much later in their school career.
